# The Prevalence of *Helicobacter pylori* Virulence Factors in Bhutan, Vietnam, and Myanmar Is Related to Gastric Cancer Incidence

**DOI:** 10.1155/2015/830813

**Published:** 2015-05-18

**Authors:** Tran Thi Huyen Trang, Seiji Shiota, Miyuki Matsuda, Tran Thanh Binh, Rumiko Suzuki, Ratha-korn Vilaichone, Varocha Mahachai, Lotay Tshering, Ho D. Q. Dung, Tomohisa Uchida, Osamu Matsunari, Thein Myint, Vu Van Khien, Yoshio Yamaoka

**Affiliations:** ^1^Department of Environmental and Preventive Medicine, Oita University Faculty of Medicine, 1-1 Idaigaoka, Hasama-machi, Yufu, Oita 879-5593, Japan; ^2^Department of Molecular Biology, 108 Hospital, Hanoi, Vietnam; ^3^Department of Endoscopy, Cho Ray Hospital, Ho Chi Minh, Vietnam; ^4^Gastroenterology Unit, Department of Medicine, Thammasat University Hospital, Pathum Thani 12120, Thailand; ^5^GI and Liver Center, Bangkok Medical Center, Bangkok 10310, Thailand; ^6^Department of Surgery, Jigme Dorji Wangchuck National Referral Hospital, Thimphu, Bhutan; ^7^Department of Molecular Pathology, Oita University Faculty of Medicine, Yufu 897-5593, Japan; ^8^Department of Gastroenterology, Oita University Faculty of Medicine, Yufu 879-5593, Japan; ^9^Department of Gastroenterology, Yangon General Hospital, Yangon 11131, Myanmar; ^10^Department of Gastroenterology, 108 Hospital, Hanoi, Vietnam; ^11^Department of Medicine-Gastroenterology, Baylor College of Medicine and Michael E. DeBakey Veterans Affairs Medical Center, 2002 Holcombe Boulevard, Houston, TX 77030, USA

## Abstract

Gastric cancer is a significant health problem in Asia. Although the prevalence of *Helicobacter pylori* infection is similar in Bhutan, Vietnam, and Myanmar, the incidence of gastric cancer is highest in Bhutan, followed by Vietnam and Myanmar. We hypothesized that *H. pylori* virulence factors contribute to the differences. The status of *cagA*, *vacA*, *jhp0562*, and *β*-*(1,3)galT(jhp0563)* was examined in 371 *H. pylori*-infected patients from Bhutan, Vietnam, and Myanmar. Each virulence factor could not explain the difference of the incidence of gastric cancer. However, the prevalence of quadruple-positive for *cagA*, *vacA* s1, *vacA* m1, and *jhp0562*-positive/*β*-*(1,3)galT*-negative was significantly higher in Bhutan than in Vietnam and Myanmar and correlated with gastric cancer incidence. Moreover, gastritis-staging scores measured by histology of gastric mucosa were significantly higher in quadruple-positive strains. We suggest that the *cagA*, *vacA* s1, *vacA* m1, and *jhp0562*-positive/*β*-*(1,3)galT*-negative genotype may play a role in the development of gastric cancer.

## 1. Introduction

Many studies indicate that the highest incidence rate of gastric cancer (GC) is found in East Asia (highest in Korea, followed by Mongolia, Japan, and China) [1]. However, the incidence of GC in South Central and South East Asia is variable and still unclear. The age standardized incidence rate (ASR) of GC is reported to be high in some countries such as Kazakhstan or Bhutan and to a lesser extent Vietnam, whilst it is lower in Myanmar and India (accessible at http://globocan.iarc.fr/). Bhutan, a small landlocked country in South Asia, has no national guidelines or recommendations for GC screening. Data from GLOBOCAN 2008 showed the ASR of GC in Bhutan (24.2 cases/100,000 per year) to be higher than that in Kazakhstan (20.6/100,000 per year), Vietnam (18.9/100,000 per year, approximately 1.28 times lower than in Bhutan), or Myanmar (11.0/100,000 per year, approximately 2.2 times lower than in Bhutan). However, four years later, data from GLOBOCAN 2012 reported that the ASR of GC in Bhutan had fallen to 17.2 cases/100,000 per year. This was still higher than the ASR of GC in Vietnam and Myanmar, but lower than that of Kazakhstan (21.6/100,000 per year). However, estimates of ASR in Bhutan are somewhat uncertain, because of the extremely low number of GC cases reported (e.g., only 92 cases in GLOBOCAN 2012). Indeed, when we performed a survey using gastroduodenal endoscopy in Bhutan in 2010, we found five cases of GC among 372 volunteers [2]. In our second survey performed in 2014, we also found six cases of GC among 470 volunteers (unpublished data). Therefore, we believe that the actual number of GC patients in Bhutan is higher than previously estimated.

The association between* H. pylori* infection and GC is well established [3, 4], but high prevalence of* H. pylori* infection is not always associated with high incidence of GC. For example, despite the high* H. pylori* infection rate in India, the incidence of GC is low, a phenomenon that has been termed the “Asian enigma” [5]. Moreover, many studies have demonstrated that virulence factors of* H. pylori* such as* cagA* and* vacA *play important roles in the severe* H. pylori* infection-mediated gastric diseases and contribute partly to the geographic variation in the ASR of GC [6–9]. Additionally, a number of recent studies indicated that the novel* H. pylori* factors* jhp0562* and *β-(1,3)galT* are associated with peptic ulcer diseases [10, 11].* Jhp0562 *encodes a glycosyltransferase involved in the synthesis of lipopolysaccharide (LPS); *β-(1,3)galT* shares a high level of sequence similarity with* jhp0562 *and is involved in the Lewis (Le) antigen expression of LPS. The presence of* jhp0562* alone (*jhp0562*-positive/*β-(1,3)galT*-negative) is associated with peptic ulcers rather than with gastritis. Our previous study indicated that the prevalence of* jhp0562*-positive/*β-(1,3)galT*-negative genotype was very high among Japanese strains and low among the US strains [10]. Therefore, together with virulence factors such as* cagA* and* vacA*,* jhp0562* and *β-(1,3)galT* might be predictors of severe clinical outcomes from* H. pylori* infection, as well as of GC.

Because the differences in GC incidence can be explained in part by differences between* H. pylori* strains [9], we aimed to examine the prevalence of* H. pylori* virulence factors (*cagA*,* vacA, jhp0562*, and *β-(1,3)galT*) in three South Asian countries with different incidences of GC: Bhutan, Vietnam, and Myanmar.

## 2. Methods

### 2.1. Patients

*H. pylori* strains were obtained from the gastric mucosa of* H. pylori*-infected subjects in Bhutan, Vietnam, and Myanmar.* H. pylori* strains were obtained in three cities (Thimphu, Punakha, and Wangdue) in Bhutan in 2010 [2, 12] and two cities (Yangon and Mandalay) in Myanmar in 2012 [13]. In Vietnam, we used samples isolated in two cities (Hanoi and Ho Chi Minh) in 2008 [7]. All the subjects from these countries were selected from our previous studies [7, 12, 13]. In each city, an endoscopy survey was performed over a continuous period of three to five days and all volunteers meeting the inclusion criteria were enrolled in this study. We included subjects greater than 16 years old with dyspeptic symptoms. Subjects with a history of partial gastric resection were excluded. Subjects who received* H. pylori* eradication therapy or treatment with antibiotics, bismuth-containing compounds, H_2_-receptor blockers, or proton pump inhibitors within four weeks prior to the study were also excluded.

Presentation included gastritis, duodenal ulcer (DU), gastric ulcer (GU), and GC. DU, GU, and GC were identified by endoscopy. Written informed consent was obtained from all participants, and the protocol was approved by the Ethics Committees of Jigme Dorji Wangchuck National Referral Hospital in Bhutan, Cho Ray Hospital and Bach Mai Hospital in Vietnam, Yangon General Hospital and Mandalay General Hospital in Myanmar, Thammasat University Hospital in Thailand, and Oita University Faculty of Medicine in Japan.

### 2.2. *H. pylori* Genotyping

Antral biopsy specimens were obtained for the isolation of* H. pylori* using standard culture methods as previously described [14].* H. pylori* DNA was extracted using the QIAamp DNA Mini Kit (QIAGEN, Valencia, CA) and used to analyze* H. pylori* genotyping. The status of* cagA*,* vacA *s1,* vacA *m1,* jhp0562*, and *β-(1,3)galT* was determined by polymerase chain reaction (PCR) as described previously [15–18]. The primer sets used are described in Table 1. PCR amplification for* jhp0562* and *β-(1,3)galT* was performed using one primer pair, 5′-TGA AAA GCC CTT TTG ATT TTG-3′ and 5′-GCT GTA GTG GCC ACA TAC ACG-3′, as described previously [18].* H. pylori* strain 26695 (ATCC 700392), which is negative for* jhp0562* and positive for *β-(1,3)galT*, and strain J99 (ATCC 700824), which is positive for both genes, were used as the reference strains [10]. The primers generated two PCR products with 301 and 602 bp in strain J99, corresponding to* jhp0562* and *β-(1,3)galT*, respectively, and only one PCR product with 558 bp in strain 26695, corresponding to *β-(1,3)galT*(*hp0619*). The amplified fragments were separated and visualized by gel electrophoresis.

### 2.3. Staging for Gastritis

All biopsy materials were fixed in 10% buffered formalin for 24 h and then embedded in paraffin. Serial sections were stained with hematoxylin and eosin. Histological analysis of the gastric mucosa was performed according to the Updated Sydney System [19]. The degree of inflammation, neutrophil activity, atrophy, intestinal metaplasia, and bacterial density was classified into four grades: 0, normal; 1, mild; 2, moderate; and 3, marked. In addition, on the basis of the topographic locations (antrum and corpus), the gastritis stage (the severity and topography of atrophy) was assessed according to the Operative Link on Gastritis Assessment (OLGA) system [20, 21].

### 2.4. Statistical Analysis

Variables such as gender, mean age, and the status of* cagA*,* vacA*,* jhp0562*, and *β-(1,3)galT* were evaluated. The chi-square test was used to examine the association between each genotype and country or clinical outcomes. A multivariate logistic regression model was used to calculate the odds ratios (OR) of the clinical outcomes by including age, sex, and the* H. pylori* genotypes. All determinants with *P* values of <0.10 were entered together in the full model of logistic regression, and the model was reduced by excluding variables with *P* values of >0.10. Spearman rank coefficients (*r*) were also determined to evaluate the association between the different genotypes of the strains. A *P* value of <0.05 was accepted as statistically significant. SPSS version 19.0 (SPSS, Inc., Chicago, IL) was used for all statistical analyses.

## 3. Results

### 3.1. Prevalence of Gastric Diseases and Virulence Factor

A total of 371* H. pylori* strains were successfully cultured: 200 from Bhutan (161 with gastritis, 18 with DU, 20 with GU, and 1 with GC), 102 from Vietnam (76 with gastritis, 14 with DU, 12 with GU, and no GC cases), and 69 from Myanmar (66 with gastritis, 1 with DU, 1 with GU, and 1 with GC). The characteristics of each study population are described in Table 2. The average age in Vietnam (44.5 ± 13.0) was significantly higher than in Myanmar (40.1 ± 11.5) or Bhutan (36.6 ± 13.8). There was no difference between the male: female ratio of patients from each country. However, in Bhutan, the percentage of male patients was significantly higher in the peptic ulcer group than in the gastritis group (71.1% versus 41.0%, *P* = 0.001). In Vietnam, the percentage of male patients was significantly higher in the peptic ulcer group (65.4% versus 39.5%, *P* = 0.02). In Myanmar, only three peptic ulcer cases were found. The distribution of the status of* cagA*,* vacA*,* jhp0562*, and *β-(1,3)galT* in the three countries is also shown in Table 2.

### 3.2. Virulence Factors of* H. pylori* and Clinical Outcomes

First, we examined the association between* H. pylori* genotypes and clinical outcomes (Table 3). In Bhutan, there were no significant differences in the presence/status of* H. pylori* virulence factors between the gastritis and peptic ulcer groups. In Vietnam, the prevalence of *β-(1,3)galT* was significantly higher in strains isolated from patients with peptic ulcer compared with gastritis patients (30.8% versus 13.2%, *P* = 0.04). The presence of* jhp0562* single-positive strains was significantly higher in the gastritis group than in the peptic ulcer group (86.8% versus 69.2%, *P* = 0.04). The prevalence of strains double-positive for* jhp0562* and *β-(1,3)galT* was significantly higher in the peptic ulcer group than in the gastritis group (26.9% versus 10.5%, *P* = 0.04). However, after adjustment for age and gender, these differences did not reach statistical significance (*P* = 0.11 for all cases). In Myanmar, peptic ulcer was found in only three cases; we therefore could not analyze the differences between gastritis and peptic ulcer for this group.

### 3.3. Gastritis Scores in Patients with Gastritis in Each Studied Country

Next, we included the strains isolated only from subjects with gastritis for histological analyses (Table 4). This group comprised 161 patients from Bhutan, 76 from Vietnam, and 66 from Myanmar. Average age was 44.1 ± 12.7 years old in Vietnam, which was significantly higher than Bhutan and Myanmar (36.8 ± 13.4 and 40.1 ± 11.7, *P* < 0.001 and *P* = 0.03, resp.). There was no difference in the male : female ratio between countries.

Patients from Bhutan showed significantly higher scores for activity, atrophy, and intestinal metaplasia in the antrum than patients from Vietnam (*P* < 0.001, <0.001, and *P* = 0.03, resp.). Inflammation in the corpus was also significantly higher in Bhutanese than in Vietnamese patients (*P* = 0.01). The atrophic score in the antrum and corpus in subjects from Bhutan was significantly higher than that in Myanmar (*P* < 0.001 and *P* = 0.001, resp.). Vietnamese subjects showed significantly higher atrophic score in the corpus than that of Myanmar (*P* < 0.001), whereas the score of activity in the antrum was significantly higher in Myanmar than Vietnam (*P* = 0.02).

Gastritis-staging scores were also classified according to the OLGA staging system. The OLGA score was significantly higher in Bhutan than Vietnam and Myanmar (*P* < 0.001 in both comparisons). There was no difference in OLGA score between Vietnam and Myanmar.

### 3.4. Virulence Factors of* H. pylori* in Patients with Gastritis in Each Studied Country

In patients with gastritis, the prevalence of* cagA*-positive strains was significantly higher in Bhutan than Vietnam and Myanmar (100% versus 94.7% and 89.4%, *P* = 0.01 and *P* < 0.001, resp.). All strains in Bhutan and Vietnam had the* vacA* s1 genotype; in Myanmar, all except for two strains had the* vacA *s1 genotype.* vacA *m1 strains were found in 75.8% of gastritis patients in Bhutan, 42.1% in Vietnam, and 87.9% in Myanmar. The prevalence of the* vacA* m1 genotype was significantly higher in Bhutan and Myanmar than Vietnam (*P* < 0.001 in both cases). The prevalence of the* vacA* m1 genotype was significantly higher in strains from Myanmar than those from Bhutan (*P* = 0.04). The prevalence of the* cagA*-positive/*vacA* s1m1 genotype was significantly higher in strains from Bhutan and Myanmar than those from Vietnam (75.8% and 80.3% versus 39.5%, *P* < 0.001 and *P* < 0.001, resp.).

*jhp0562*-positive strains were found in 98.1% of gastritis patients in Bhutan, 97.4% in Vietnam, and 97.0% in Myanmar. There was no difference in the* jhp0562*-positive rate among the three countries, but the prevalence of *β-(1,3)galT*-positive strains was significantly higher in Myanmar (71.2%) than in Bhutan (18.0%) or Vietnam (13.2%) (*P* < 0.001 in both cases). The prevalence of strains double-positive for* jhp0562* and *β-(1,3)galT* was also significantly higher in Myanmar (68.2%) than in Bhutan (16.1%) or Vietnam (10.5%) (*P* < 0.001 in both cases). There were no significant differences in the prevalence of the* jhp0562*-negative/*β-(1,3)galT*-positive genotype between the three countries (1.9% in Bhutan, 2.6% in Vietnam, and 3.0% in Myanmar). The prevalence of strains quadruple-positive for* cagA*,* vacA* s1,* vacA* m1, and* jhp0562*-positive/*β-(1,3)galT*-negative was 62.1% in Bhutan, which was significantly higher than Vietnam (34.2%) and Myanmar (22.7%) (*P* < 0.001 in both cases). Furthermore, the presence of quadruple-positive status was significantly correlated with OLGA score (*P* < 0.0001). The OLGA score (mean [median]) was significantly higher in quadruple-positive than other types of strains (1.41 [1] versus 1.12 [1], *P* < 0.0001).

### 3.5. Correlations between* cagA*,* jhp0562*, and *β-(1,3)galT*

In Bhutan, all cases were* cagA*-positive. In Vietnam, a positive correlation between the presence of* cagA* and* jhp0562* was observed (*r* = 0.498, *P* < 0.001). On the other hand, there was no correlation between the presence of* cagA* and *β-(1,3)galT* in Vietnam (*P* = 0.18). In Myanmar, there were also no correlations between* cagA*,* jhp0562*, and *β-(1,3)galT* (*P* = 0.08 and *P* = 0.50, resp.).

## 4. Discussion

*Jhp0562 *has been reported to be associated with peptic ulcer diseases in children, but not in adults, in the Portuguese population [18]. In a subsequent study in Portuguese children by the same group, the presence of* jhp0562* alone (*jhp0562*-positive/*β-(1,3)galT-*negative) was associated with peptic ulcers, whereas the presence of *β-(1,3)galT* alone (*jhp0562*-negative/*β-(1,3)galT-*positive) was associated with gastritis [11]. We previously reported that the prevalence of* jhp0562* was higher in Japan than in the USA (100% versus 78%) [10]. Furthermore, we reported that the* jhp0562*-positive/*β-(1,3)galT*-negative genotype was significantly associated with peptic ulcer in the USA [10]. In the present study, we found that almost all strains isolated in Bhutan, Vietnam, and Myanmar were positive for jhp0562 (98.5%, 97.1%, and 97.2%, resp.).* H. pylori *strains carrying* cagA* and the potentially toxigenic* vacA* s1 alleles were found to predominate in all studied countries, but strains carrying the* vacA* m1 were higher in Bhutan and Myanmar than in Vietnam. We did not identify any relationship between the prevalence of any studied virulence factors and the difference of GC incidences in Bhutan, Myanmar, and Vietnam. It might be similar to the other East Asian countries, where* cagA*-positive were found in almost all strains and* vacA* s1m1 is dominant; therefore, these virulence factors could not show the usefulness as markers of GC. Interestingly, we found the contribution of novel factors* jhp0562* and *β-(1,3)galT* in describing the different incidences of GC between Bhutan, Vietnam, and Myanmar. The prevalence of strains quadruple-positive for* cagA*,* vacA* s1,* vacA* m1, and* jhp0562*-positive/*β-(1,3)galT*-negative was the highest in Bhutan, followed by Vietnam and Myanmar, which correlated with the incidence of GC reported in GLOBOCAN 2012 (http://globocan.iarc.fr/). Furthermore, the presence of the quadruple-positive genotype was significantly correlated with the OLGA score. These results suggest that the prevalence of strains quadruple-positive for* cagA*,* vacA* s1,* vacA* m1, and* jhp0562*-positive/*β-(1,3)galT*-negative might be a marker for the development of GC. Our finding proved the essential element of virulence factors over* H. pylori* infection in evaluating the higher incidence of GC between the countries with the same* H. pylori* prevalence. The limitation of the study is that only very few strains were obtained from GC; therefore, the true prevalence of these quadruple-positive factors in GC is difficult to estimate.

In this study, there were no differences in the prevalence of* H. pylori* virulence factors including* cagA*,* vacA*,* jhp0562*, and *β-(1,3)galT *between the gastritis and peptic ulcer groups from Bhutan. However, the prevalence of the* cagA*-positive,* vacA* s1m1, and* jhp0562*-positive/*β-(1,3)galT*-negative genotype was higher in Bhutan than in Vietnam or Myanmar. In addition, all* cagA*-positive strains in Bhutan were also positive for* jhp0562*. The prevalence of* jhp0562* was significantly positively correlated with* cagA* in Vietnam. In our previous study, the prevalence of* jhp0562* in the* cagA*-positive strains was 100% in Japan, and the prevalence of* jhp0562* was strongly associated with that of* cagA* in the US population [10].* cagA* status is also linked to the* vacA* s region type, and it is further closely linked to the presence of* babA* and* oipA* “on” status, which are virulence factors coding for outer membrane proteins [22–24]. As a result, almost all* H. pylori* strains circulating in Japan are extremely virulent, harboring the* cagA*,* vacA* s1 genotype,* oipA *“on” status, and* babA* irrespective of clinical outcomes [24–26]. Therefore,* H. pylori *strains isolated from Bhutan can also be considered highly virulent. Thus, we suggested that the phenotype resulting from the expression of* cagA*,* vacA* s1m1, and* jhp0562* confers a biological advantage to the strains, with the cumulative action of each factor contributing simultaneously to the fitness of the strains* in vivo* and a more pronounced proinflammatory response. It might be better to hypothesize that these factors interact synergistically with each other and induce serious diseases than to discuss which of these factors is the most virulent. Our suggestion might partly explain the correlation between the higher prevalence of strains with quadruple-positive virulence and increased GC incidence.

It remains unclear whether the presence of the* jhp0562*-positive/*β-(1,3)galT*-negative genotype is associated with higher gastritis score.* jhp0562* encodes a glycosyltransferase involved in the synthesis of the chemical structure of LPS.* H. pylori* expresses Le antigens in its LPS, both type 1 (Le^a^ and Le^b^) and type 2 (Le^x^ and Le^y^); these are structurally related to the human blood group antigens also expressed in gastric epithelial cells [27, 28].* jhp0562* is located immediately upstream of *β-(1,3)galT*, which in turn encodes a *β-(1,3)* galactosyltransferase involved in type I Le antigen synthesis [29, 30]. The* jhp0562* and *β-(1,3)galT* genes are highly similar (>80%), especially at their 5′ and 3′ ends [31]. Although these Le antigenic structures were reported to be important for bacterial colonization, adhesion, and evasion of host immune response [32–34], the role of these in* H. pylori* infection has not been elucidated. A recent study reported that mutagenesis of* jhp0562* resulted in the loss of expression of all Le types, suggesting that the product of this gene is truly an essential glycosyltransferase for Le expression [31]. These successive complementation results showed that *β-(1,3)galT* alone was insufficient for type 1 Le synthesis and that* jhp0562* must also be present.* jhp0562* contributed to both type 1 and type 2 Le synthesis, while *β-(1,3)galT* was essential only for type 1 Le synthesis. Another report revealed that East Asian strains express types 1 and 2 Le antigens, whereas Western strains express only type 2 Le antigens [35]. It has also been shown that the expression of Le^x^ or Le^y^ in* H. pylori* isolates is significantly higher in peptic ulcer than in nonulcer dyspepsia patients [36]. On the other hand, another study in China, where most strains express Le^x^ or Le^y^, did not confirm this relationship [37]. This is similar to* cagA*, which cannot be used as a marker in areas where the incidence of GC is high.* In vitro* and* in vivo* studies are necessary to elucidate the causal relationship between* jhp0562* and *β-(1,3)galT* and to investigate the mechanisms by which these gene products correlate with clinical outcomes.

## 5. Conclusion

In conclusion, each virulence factor including* cagA*,* vacA* s1, m1,* jhp0562, *and *β-(1,3)galT* could not explain the difference of the incidence of GC between Bhutan, Vietnam, and Myanmar. Interestingly, the prevalence of the quadruple-positive genotype for* cagA*,* vacA* s1,* vacA* m1, and* jhp0562*-positive/*β-(1,3)galT*-negative was highest in Bhutan, followed by Vietnam and Myanmar, which correlated with the incidence of GC. Furthermore, the presence of the quadruple-positive genotype was significantly correlated with severe gastritis. This suggests that the prevalence of the quadruple-positive genotype for* cagA*,* vacA* s1,* vacA* m1, and* jhp0562*-positive/*β-(1,3)galT*-negative might be a marker for the development of GC.

## Figures and Tables

**Table 1:** Primer sequences.

Genes	Primer sequences (5′→3′)	PCR product (bp)	PCR conditions
*cagA *	ACC CTA GTC GGT AAT GGG	521	94°C, 1 min; 52°C, 1 min; 72°C, 1 min (35 cycles)
	GCT TTA GCT TCT GAY ACY GC^*^		

*vacA *			
s1/s2	ATG GAA ATA CAA CAA ACA CAC	259/268	94°C, 1 min; 52°C, 1 min; 72°C, 1 min (35 cycles)
	CTG CTT GAA TGC GCC AAA C		
m1/m2	CAA TCT GTC CAA TCA AGC GAG	567/642	
	GCG TCA AAA TAA TTC CAA GG		

*jhp0562*/*β-(1,3)galT *	TGA AAA GCC CTT TTG ATT TTG	301/602	95°C, 30 s; 56°C, 30 s; 72°C, 1 s (35 cycles)
	GCT GTA GTG GCC ACA TAC ACG		

^*^Y = C + T.

**Table 2:** Characteristics of *Helicobacter pylori*-infected patients in Bhutan, Vietnam, and Myanmar.

	Bhutan		Vietnam		Myanmar	
*n*	200		102		69	
Mean age	36.6 ± 13.8^*^^†^		44.5 ± 13.0		40.1 ± 11.5^**^	
Male	94	(47.0%)	47	(46.1%)	28	(40.6%)
Gastric cancer	1	(0.5%)	0	(0.0%)	1	(1.4%)
Peptic ulcer	38	(19.0%)	26	(25.5%)	2	(2.9%)
Gastritis	161	(80.5%)	76	(74.5%)	66	(95.7%)
*cagA *	200	(100.0%)^*^^†^	97	(95.1%)	61	(88.4%)^**^
*vacA* s1	200	(100.0%)	102	(100.0%)	67	(97.1%)
*vacA* m1	154	(77.0%)^*^^†^	48	(47.1%)	61	(88.4%)^**^
*jhp0562*-positive	197	(98.5%)	99	(97.1%)	67	(97.1%)
*β-(1,3)galT*-positive	34	(17.0%)^†^	18	(17.6%)	50	(72.5%)^**^
*jhp0562-*positive/*β-(1,3)galT*-negative	166	(83.0%)^†^	84	(82.4%)	19	(27.5%)^**^
*jhp0562*-negative/*β-(1,3)galT*-positive	3	(1.5%)	3	(2.9%)	2	(2.9%)
Double-positive of *jhp0562* and *β-(1,3)galT *	31	(15.5%)^†^	15	(14.7%)	48	(69.6%)^**^
*cagA*/*vacA* s1m1	154	(77.0%)^*^	46	(45.1%)	55	(79.7%)^**^
*cagA*/*vacA* s1m1/*jhp0562*-positive/*β-(1,3)galT*-negative	128	(64.0%)^*^^†^	37	(36.3%)	15	(21.7%)^**^

∗ indicates a statistically significant difference between Bhutan and Vietnam.

∗∗ indicates a statistically significant difference between Myanmar and Vietnam.

† indicates a statistically significant difference between Bhutan and Myanmar.

**(a):** 

Bhutan	Gastritis		Peptic ulcer	
*n*	161		38	
Mean age	36.8 ± 13.4		35.3 ± 15.3	
Male	66	(41.0%)	27	(71.1%)^*^
*cagA *	161	(100.0%)	38	(100.0%)
*vacA* s1	161	(100.0%)	38	(100.0%)
*vacA* m1	122	(75.8%)	31	(81.6%)
*jhp0562*-positive	158	(98.1%)	38	(100.0%)
*β-(1,3)gal-* positive	29	(18.0%)	4	(10.5%)
*jhp0562*-positive/*β-(1,3)galT-*negative	132	(82.0%)	34	(89.5%)
*jhp0562*-negative/*β-(1,3)galT*-positive	3	(1.9%)	0	(0.0%)
Double-positive of *jhp0562* and *β-(1,3)galT *	26	(16.1%)	4	(10.5%)
*cagA*/*vacA* s1m1	122	(75.8%)	31	(81.6%)
*cagA*/*vacA* s1m1/*jhp0562*-positive/*β-(1,3)galT*-negative	100	(62.1%)	28	(73.7%)

∗ indicates *P* < 0.05.

**(b):** 

Vietnam	Gastritis		Peptic ulcer	
*n*	76		26	
Mean age	44.1 ± 12.7		46.0 ± 14.1	
Male	30	(39.5%)	17	(65.4%)^*^
*cagA *	72	(94.7%)	25	(96.2%)
*vacA* s1	76	(100.0%)	26	(100.0%)
*vacA* m1	32	(42.1%)	16	(61.5%)
*jhp0562*-positive	74	(97.4%)	25	(96.2%)
*β-(1,3)galT*-positive	10	(13.2%)	8	(30.8%)^*^
*jhp0562*-positive/*β-(1,3)galT*-negative	66	(86.8%)	18	(69.2%)^*^
*jhp0562*-negative/*β-(1,3)galT*-positive	2	(2.6%)	1	(3.8%)
Double-positive of *jhp0562* and *β-(1,3)galT *	8	(10.5%)	7	(26.9%)^*^
*cagA*/*vacA* s1m1	30	(39.5%)	16	(61.5%)
*cagA*/*vacA* s1m1/*jhp0562*-positive/*β-(1,3)galT*-negative	26	(34.2%)	11	(42.3%)

∗ indicates *P* < 0.05.

**Table 4:** *Helicobacter pylori* virulence factors and gastritis scores in patients with gastritis in three countries.

	Bhutan		Vietnam		Myanmar	
*n*	161		76		66	
Mean age	36.8 ± 13.4^*^^†^		44.1 ± 12.7		40.1 ± 11.7^**^	
Male	66	(41.0%)	30	(39.5%)	27	(40.9%)
*cagA *	161	(100.0%)^*^^†^	72	(94.7%)	59	(89.4%)
*vacA* s1	161	(100.0%)	76	(100.0%)	64	(97.0%)
*vacA* m1	122	(75.8%)^*^^†^	32	(42.1%)	58	(87.9%)^**^
*jhp0562*-positive	158	(98.1%)	74	(97.4%)	64	(97.0%)
*β-(1,3)galT*-positive	29	(18.0%)^†^	10	(13.2%)	47	(71.2%)^**^
*jhp0562*-positive/*β-(1,3)galT*-negative	132	(82.0%)^†^	66	(86.8%)	19	(28.8%)^**^
*jhp0562*-negative/*β-(1,3)galT*-positive	3	(1.9%)	2	(2.6%)	2	(3.0%)
Double-positive of *jhp0562* and *β-(1,3)galT *	26	(16.1%)^†^	8	(10.5%)	45	(68.2%)^**^
*cagA*/*vacA* s1m1	122	(75.8%)^*^	30	(39.5%)	53	(80.3%)^**^
*cagA*/*vacA* s1m1/*jhp0562*-positive/*β-(1,3)galT*-negative	100	(62.1%)^*^^†^	26	(34.2%)	15	(22.7%)
Antrum						
Activity	1.53 (1)^*^		1.20 (1)		1.44 (1)^**^	
Inflammation	1.76 (2)		1.67 (2)		1.65 (2)	
Atrophy	1.42 (1)^*^^†^		0.89 (1)		0.89 (1)	
Intestinal metaplasia	0.16 (0)^*^		0.04 (0)		0.14 (0)	
Corpus						
Activity	0.93 (1)		0.92 (1)		0.88 (1)	
Inflammation	1.14 (1)^*^		1.23 (1)		1.08 (1)^**^	
Atrophy	0.55 (0)^†^		0.58 (1)		0.26 (0)^**^	
Intestinal metaplasia	0.02 (0)		0.05 (0)		0.00 (0)	
OLGA	1.51 (1)^*^^†^		1.01 (1)		0.94 (1)	

∗ indicates a statistically significant difference between Bhutan and Vietnam.

∗∗ indicates a statistically significant difference between Myanmar and Vietnam.

† indicates a statistically significant difference between Bhutan and Myanmar.

For histological scores (minimum 0 to maximum 3) and OLGA score (minimum 0 to maximum 4), mean (median) is presented.

## Abbreviations

ASR: Age standardized incidence rate
DU: Duodenal ulcer
GC: Gastric cancer
LPS: Lipopolysaccharide
PCR: Polymerase chain reaction.
